# Modifiable glymphatic-related brain clearance physiology in humans: a critical narrative review of interventional evidence

**DOI:** 10.3389/fnins.2026.1984401

**Published:** 2026-10-09

**Authors:** Marta M. Wrzesień, Kamil Pobozy, Grzegorz Turek, Wojciech Czyzewski, Binbin Zhu, Mirosław Ząbek

**Affiliations:** 1 Medical University of Warsaw, Warsaw, Poland; 2 Department of Neurosurgery, Mazovian Bródnowski Hospital, Warsaw, Poland; 3 Department of Neurosurgery, Maria Sklodowska-Curie National Research Institute of Oncology, Warsaw, Poland; 4 Department of Didactics and Medical Simulation, Medical University of Lublin, Lublin, Poland; 5 Anesthesiology Department, The First Affiliated Hospital of Ningbo University, Ningbo, China; 6 Gamma Knife Center, Warsaw, Poland; 7 Department of Neurosurgery, Center of Postgraduate Medical Education, Warsaw, Poland

**Keywords:** brain clearance, cerebrospinal fluid, glymphatic system, neurofluid dynamics, neuroimaging, perivascular space, sleep, solute transport

## Abstract

The glymphatic model proposes that cerebrospinal fluid exchanges with interstitial fluid along perivascular pathways, potentially facilitating brain solute clearance. Interest in whether this physiology can be modified has prompted human studies of sleep, exercise, respiratory manipulation, circadian interventions, and neuromodulation, but these studies use endpoints that differ substantially in biological directness. This critical narrative review evaluates human interventional and longitudinal evidence for modifiable glymphatic-related brain-clearance physiology and distinguishes altered neurofluid dynamics from evidence of solute transport. Sleep provides the most convincing convergent evidence: total sleep deprivation impairs intrathecal tracer clearance, whereas normal sleep increases the overnight appearance of amyloid-β and tau in plasma. Sustained aerobic exercise modifies contrast-based brain influx and meningeal lymphatic measures, although evidence remains preliminary. Slow deep breathing and intermittent hypercapnia alter cerebrospinal fluid dynamics; hypercapnia also increases several plasma brain-protein concentrations, but enhanced clearance is unconfirmed. Bright light therapy and transcranial magnetic stimulation modify diffusion-based perivascular imaging proxies that remain indirect and biologically nonspecific. Human neurofluid physiology may therefore be modifiable, but changes in fluid motion or surrogate imaging signals should not be equated with enhanced solute clearance. Clinically meaningful modulation will require controlled studies linking mechanistic target engagement to direct solute transport and neurological outcomes.

## 1 Introduction

The brain continuously produces metabolic waste products that must be removed to maintain a stable extracellular environment. The glymphatic model describes one possible route for this clearance. According to this model, cerebrospinal fluid (CSF) enters spaces surrounding cerebral arteries, exchanges with interstitial fluid, and supports the movement of dissolved substances through brain tissue toward drainage pathways. Astrocytes, a major type of glial cell, are thought to facilitate this process through their end-feet, which closely surround cerebral blood vessels (Figure 1). This concept provided a plausible explanation for how soluble metabolites might be transported out of the brain (Iliff et al., 2012).

**Figure 1:**
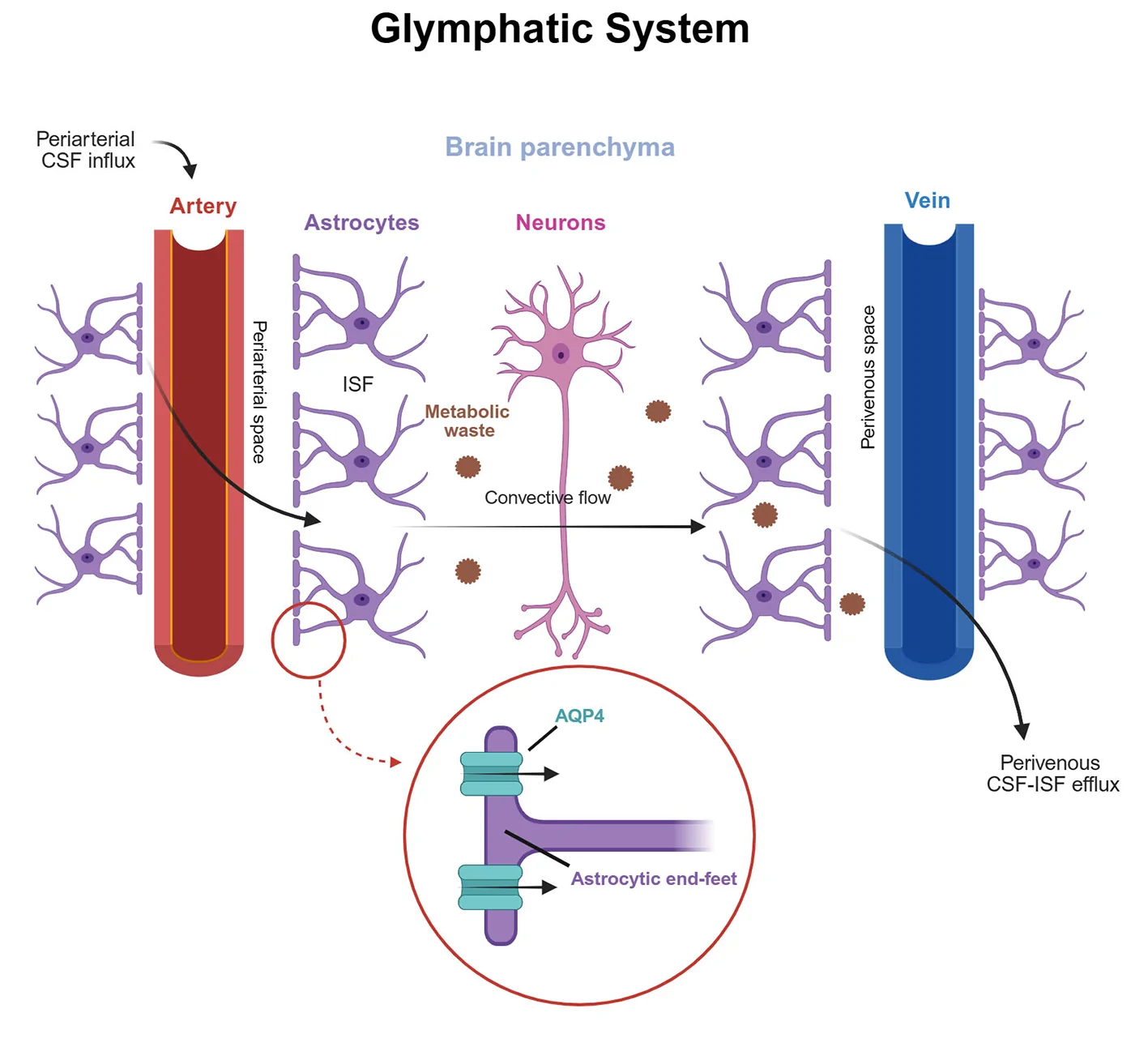
Conceptual representation of the glymphatic model. Cerebrospinal fluid (CSF) enters along periarterial spaces, exchanges with interstitial fluid (ISF) within the brain parenchyma, and may support solute transport toward perivenous pathways. Aquaporin-4 (AQP4) water channels on astrocytic endfeet are proposed to facilitate this exchange. Created in BioRender. Poboży, K. (2026) https://BioRender.com/fhl07kg.

The current understanding of brain-fluid transport is broader than the original glymphatic model. It includes periarterial and perivenous spaces, movement of fluid and solutes through the interstitial compartment, CSF production and turnover, exchange across the blood-brain and blood-CSF barriers, and drainage through meningeal lymphatic vessels. These processes are related but are not biologically equivalent and should not be regarded as components of a single, uniform pathway. The principal controversies concern the direction and magnitude of fluid movement, the forces that drive it, and the relative contribution of each transport route under physiological and pathological conditions (Hladky and Barrand, 2022; Mestre et al., 2020). The term glymphatic system is most appropriately used to describe perivascular and interstitial fluid exchange within brain tissue rather than all mechanisms of cerebral fluid transport such as meningeal lymphatic vessels drainage (Louveau et al., 2015; Aspelund et al., 2015).

Research interest has increasingly shifted from describing glymphatic pathways to determining whether they can be modified. Sleep (Xie et al., 2013), physical exercise (Yoo et al., 2025), controlled breathing (Lim et al., 2025), body position (Lee et al., 2015), positive airway pressure (Ozturk et al., 2023), light therapy (Chen et al., 2026), and neuromodulation (Gao et al., 2025; Zhang et al., 2025; Sundman et al., 2025) have all been proposed as interventions that might influence glymphatic-related physiology. The clinical rationale is important: impaired clearance could potentially contribute to the accumulation of amyloid-β, tau, α-synuclein, inflammatory mediators, and other substances associated with neurological disease. If an intervention could restore their removal, it might affect disease mechanisms rather than merely produce a physiological association (Rasmussen et al., 2018; Lohela et al., 2022).

However, human studies use markedly different outcomes to assess these effects. Some measure CSF velocity or displacement, whereas others assess directional water diffusion, contrast-agent exchange between CSF and brain tissue, or the appearance of brain-derived proteins in blood. These measurements represent different stages of the transport process and are not interchangeable. An increase in CSF movement does not necessarily demonstrate greater transport through the brain parenchyma. Similarly, increased water diffusion along a particular anatomical direction does not prove that waste products have been removed. Changes in plasma biomarker concentrations may reflect altered production, release, barrier permeability, distribution volume, or peripheral metabolism in addition to cerebral clearance. Consequently, the term “improved glymphatic function” may overstate what an individual study has actually demonstrated.

This review therefore addresses a focused question: which interventions have been shown to modify glymphatic-related brain-clearance physiology in humans, and what conclusions are justified by the endpoints used? Its purpose is not to rank lifestyle recommendations or propose a new clinical treatment. Instead, it aims to organize the available evidence according to the biological directness of each measurement, distinguish changes in fluid dynamics from evidence of solute clearance, identify interventions with credible human target engagement, and define the studies required before any “glymphatic therapy” can be considered clinically actionable.

## 2 Narrative review methods

### 2.1 Search strategy and scope

We conducted a focused narrative search of PubMed/MEDLINE for studies published up to 26 September 2026. The reference lists of relevant human neurofluid reviews and primary studies were also examined to identify additional publications. Search terms covered two broad domains. The first comprised concepts related to brain-fluid transport, including “glymphatic,” “perivascular,” “brain clearance,” “CSF–interstitial fluid exchange,” “meningeal lymphatic,” “DTI-ALPS,” “CSF flow,” and “blood-oxygen-level-dependent (BOLD)–CSF coupling”. The second comprised potentially modifiable factors and interventions, including sleep and sleep deprivation, exercise, breathing, hypercapnia, positive airway pressure, body position, circadian and light-based interventions, transcranial magnetic stimulation, vagus nerve stimulation, photobiomodulation, and sensory stimulation.

### 2.2 Study selection and prioritization

We prioritized human studies that evaluated whether an intervention or change in physiological state modified a glymphatic-related outcome. These included randomized and non-randomized interventional studies, crossover experiments, prospective longitudinal studies, and target-engagement studies. Observational studies were included when they provided relevant information about reversibility, temporal change, or a clinically meaningful disease trajectory.

Preclinical studies were used selectively. Animal experiments were included when no corresponding human interventional evidence was available or when they investigated a mechanism that could not be directly examined in humans. They were used to establish biological plausibility or identify translational gaps, rather than as evidence of clinical efficacy.

Two relevant preprints were included (Dagum et al., 2026a,b; Tong et al., 2026)—these studies have not yet been peer-reviewed, and should be interpreted provisionally.

Because the purpose of this review was to provide a critical conceptual synthesis, we did not use systematic-review procedures such as protocol-based screening, duplicate study selection, duplicate data extraction, or formal risk-of-bias instruments. The review should therefore not be interpreted as an exhaustive assessment of the literature or as a quantitative estimate of intervention effects.

### 2.3 Data extraction and evidence appraisal

For each included study, we considered the study design, population, intervention or exposure, comparator, measured endpoint, direction of the reported effect, and the strongest interpretation supported by that endpoint. Particular attention was given to distinguishing measurements of fluid dynamics or tissue-water diffusion from measurements that more directly reflected solute transport or clearance.

Evidence was appraised across four domains: experimental control, biological directness of the endpoint, independent replication, and linkage to a clinically meaningful outcome. Experimental control reflected the extent to which the study design could support causal inference. Endpoint directness described how closely the measured outcome represented net movement of a tracer or endogenous solute between biological compartments. Replication referred to confirmation in an independent study or population, whereas clinical linkage assessed whether the physiological change was associated with symptoms, cognition, or disease progression.

The terms direct, near-direct, intermediate, and indirect were used to describe the interpretive proximity of each endpoint to net solute transport. These categories constituted a conceptual framework developed for this review and should not be regarded as a formally validated evidence-grading system.

Generative AI assistance during final manuscript preparation was limited to language editing, organization, reference-style conversion, and document formatting; it was not used for study identification, selection, data extraction, or evidence appraisal. Full tool details are provided in the Generative AI statement.

## 3 What counts as modification of brain clearance?

Brain clearance is a multistep process, and an intervention can influence this process at several biologically distinct levels. These levels should not be treated as equivalent. A change in a mechanism that may drive fluid movement does not necessarily result in greater exchange between CSF and brain tissue. Similarly, increased fluid movement does not by itself demonstrate that a waste product has been removed from the brain.

At the most upstream level, an intervention may alter a physiological driver of neurofluid transport. Potential drivers include arterial pulsatility, slow changes in vascular diameter known as vasomotion, venous and respiratory pressure gradients, arousal state, and the volume of the extracellular space. Such changes may create conditions that favor fluid transport, but they provide evidence of mechanistic target engagement rather than clearance itself.

At the next level, an intervention may change a measurable neurofluid signal, such as CSF velocity, displacement, pulsatility, or coupling between neural, vascular, and CSF signals. These measurements demonstrate that fluid dynamics have been modified. However, they are usually obtained from the ventricles, foramen magnum, or other relatively large CSF spaces and may not reflect fluid movement through the brain parenchyma. A change in these signals should therefore be interpreted as altered neurofluid physiology rather than direct evidence of waste removal.

Stronger evidence is provided when a study demonstrates exchange between CSF and brain tissue or tracks the movement of a defined substance between biological compartments. Contrast-enhanced imaging can show the entry, distribution, and washout of a tracer within CSF and brain tissue. This is more directly related to transport, although the behavior of an administered contrast agent may not be identical to that of endogenous metabolic waste. The most informative endpoints follow a defined tracer or a brain-derived solute from its compartment of origin toward a downstream compartment, such as CSF or blood. Even these measurements require careful interpretation because the observed concentration may also be influenced by solute production, barrier permeability, dilution, and systemic elimination.

The final level is clinical relevance. Demonstrating that an intervention changes a transport endpoint does not establish that the change improves symptoms, cognition, or disease progression. The strongest evidence would come from a study showing, in the same participants, that an intervention affects its intended physiological target, modifies a validated measure of solute transport, and produces a clinically meaningful benefit. A prespecified mediation analysis would then be required to determine whether the improvement in the clinical outcome was actually explained by the change in transport. Most available human studies evaluate only one part of this sequence. Figure 2 summarizes the resulting translational chain.

**Figure 2:**
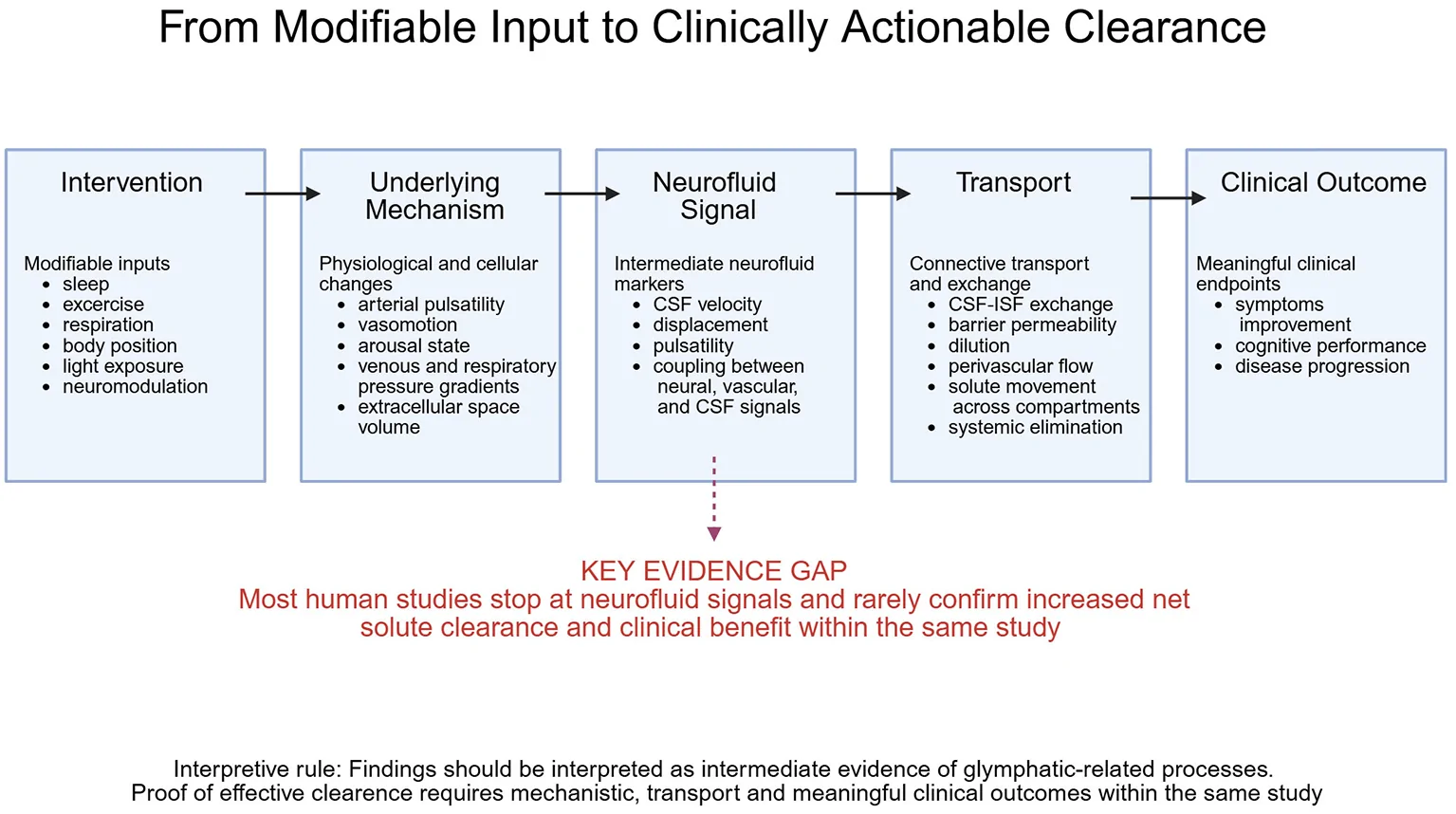
Translational evidence chain from intervention to clinically actionable clearance. Most human studies demonstrate proximal physiological or neurofluid changes; few directly measure solute transport, and none has shown that a transport change mediates a clinical benefit. CSF, cerebrospinal fluid; ISF, interstitial fluid. Created in BioRender. Poboży, K. (2026) https://BioRender.com/taattb0.

For this review, an intervention was considered to modify glymphatic-related physiology when it altered a relevant driver or neurofluid signal. A stronger claim of modified brain clearance required evidence of tracer or endogenous solute movement across biological compartments. Physiological and imaging measures were accepted as surrogate endpoints only when they were clearly described as such. Changes in CSF flow or blood-oxygen-level-dependent (BOLD)–CSF coupling were therefore interpreted as evidence of altered neurofluid dynamics. Changes in diffusion tensor imaging analysis along the perivascular space (DTI-ALPS) were interpreted as changes in directional water diffusion within selected periventricular white-matter regions. Neither finding, when considered alone, establishes increased net removal of waste from the brain. This distinction is the principal interpretive rule applied throughout this review.

## 4 Measuring brain clearance in humans: what each method can show

No single method directly measures the entire process of brain clearance in humans. Available techniques assess different stages, from tracer movement between compartments to indirect changes in water diffusion or tissue structure. The interpretive ceiling of each method is the strongest conclusion that its endpoint can support.

Intrathecal contrast-enhanced magnetic resonance imaging (MRI) provides the closest available human measure of macroscopic tracer transport. After a contrast agent is administered into the CSF, serial MRI tracks its distribution and disappearance over several h or days. This approach has demonstrated brain-wide tracer enrichment and clearance (Ringstad et al., 2018), as well as impaired clearance after total sleep deprivation (Eide et al., 2021). Its main advantage is the ability to follow a known substance across anatomical compartments. However, the procedure is invasive, intrathecal gadolinium remains off-label in many jurisdictions, and participants undergoing lumbar puncture may not represent the general population. Moreover, changes in MRI signal reflect a combination of diffusion, pressure-driven movement, dispersion, dilution, and tissue retention rather than clearance alone.

A near-direct alternative is to measure brain-derived proteins in blood. In a randomized crossover study of 39 participants, normal sleep was followed by higher morning plasma concentrations of several amyloid-β and tau species than sleep deprivation. Physiological modeling attributed part of this difference to sleep-associated transport from the brain toward the blood (Dagum et al., 2026a,b). These biomarkers are clinically relevant, but their plasma concentrations also depend on protein production, barrier permeability, plasma volume, peripheral metabolism, and systemic elimination. They therefore provide evidence consistent with brain-to-blood transport but do not represent a pure measurement of clearance.

Intravenous contrast-enhanced T1 mapping is less invasive and can estimate delayed contrast exchange between blood, CSF, and brain tissue. A prospective crossover study identified sleep-associated differences in putative glymphatic activity using this method (Lee et al., 2021). However, only a small proportion of intravenously administered contrast reaches the CSF. The signal is also affected by vascular contrast, blood-brain and blood-CSF barrier permeability, renal elimination, and imaging time. This technique is therefore best described as a measure of contrast-based CSF-tissue exchange rather than direct waste clearance.

Physiological imaging methods assess fluid movement and its potential driving forces. Fast functional MRI during sleep has demonstrated coordinated changes in neural activity, cerebral hemodynamics, and CSF inflow (Fultz et al., 2019). Phase-contrast and velocity-encoded MRI can directly measure CSF velocity and displacement and have shown that respiration influences human CSF movement (Dreha-Kulaczewski et al., 2015; Kollmeier et al., 2022; Wright et al., 2024). These methods provide valuable information about neurofluid dynamics, but measurements are usually obtained from the ventricles, cerebral aqueduct, foramen magnum, or other large CSF spaces. Greater CSF motion at these sites does not necessarily indicate increased entry into brain tissue or removal of a solute. Their appropriate interpretation is therefore altered CSF motion or neurovascular–CSF coupling, not clearance itself (Naganawa et al., 2024).

Recent advances have also improved the spatial characterization of CSF mobility within perivascular compartments. Fultz et al. (2026) used CSF-STREAM (CSF-Selective T2-prepared REadout with Acceleration and Mobility-encoding), a non-invasive MRI technique designed to isolate CSF signal from blood and tissue while providing high-resolution information on CSF mobility (Fultz et al., 2026). In healthy participants, CSF mobility was higher immediately adjacent to the middle and anterior cerebral arteries and declined sharply toward the surrounding subarachnoid space, providing imaging evidence consistent with a spatially distinct perivascular subarachnoid compartment. This approach is important because it moves beyond measurements of CSF motion in large spaces and permits characterization of the spatial organization of fluid dynamics around cerebral vessels. However, CSF-STREAM measures fluid mobility rather than solute transport and therefore does not by itself establish glymphatic clearance. Its principal value at present is as a more anatomically resolved physiological readout that may enable future studies to link perivascular CSF dynamics with tracer or endogenous-solute transport.

Diffusion tensor imaging analysis along the perivascular space (DTI-ALPS) is the widely used non-invasive proxy. It measures directional water diffusion in selected white-matter regions near the lateral ventricles, based on the assumption that diffusion along one anatomical axis partly reflects water movement near medullary perivascular spaces (Taoka et al., 2017). The index can be reproducible when acquisition and region placement are standardized (Taoka et al., 2022), but its biological specificity is limited. Crossing fibers can increase the index (Georgiopoulos et al., 2024), and normal white-matter geometry can produce similar directional asymmetry without perivascular flow (Schilling et al., 2025). Furthermore, DTI-ALPS shows only limited agreement with intrathecal tracer enrichment and clearance (Mossige et al., 2026). It should therefore be described as a perivascular-region diffusion proxy rather than a stand-alone measure of glymphatic clearance (Haller et al., 2024; Ringstad, 2024).

Structural markers, including visible perivascular spaces, choroid plexus volume, free-water measures, and parasagittal enhancement, provide anatomical context but do not measure transport (Naganawa et al., 2024; Zhao et al., 2022) . A wearable system combining electrical impedance spectroscopy, electroencephalography, and cardiovascular monitoring offers a time-resolved measure of brain parenchymal resistance. Overnight changes in resistance have been associated with sleep characteristics and contrast-based exchange (Dagum et al., 2025). Although this approach may be useful for repeated home monitoring, it reflects a physiological state related to extracellular water content rather than the movement of a defined solute.

Overall, the most direct methods are invasive and difficult to repeat, whereas the most accessible methods are less biologically specific. Confidence is greatest when an intervention produces consistent changes across complementary measurements, including a physiological driver, a transport-related endpoint, and the movement of a defined solute. Table 1 summarizes the strengths, limitations, and interpretive ceiling of each method.

**Table 1:** Human methods for assessing glymphatic-related physiology: signal, strength, and interpretive ceiling.

Method	Primary signal	Main strength	Key limitations	Interpretive ceiling
Intrathecal contrast-enhanced MRI	Serial tracer enrichment and washout in CSF and tissue	Explicit CSF tracer input; spatial kinetics over h–days	Invasive; off-label contrast; selected clinical populations; sparse sampling	Macroscopic tracer exchange/clearance under study conditions
Overnight brain-to-plasma proteins	Change in plasma amyloid-β/tau or other brain-enriched proteins	Clinically relevant endogenous solutes; scalable sampling	Integrates production, barrier transport, plasma volume, peripheral sources, and systemic clearance	Net appearance in blood; clearance attribution requires modeling or paired compartments
Intravenous contrast T1 mapping	Delayed contrast-related T1 change in CSF and parenchyma	Less invasive; repeated whole-brain imaging	Small CSF contrast fraction; vascular/barrier confounding; timing-sensitive	Putative CSF–tissue contrast exchange
Phase-contrast/velocity MRI	CSF velocity, displacement, or directional bias	Direct real-time fluid-motion measurement	Usually large CSF spaces; no parenchymal or solute endpoint	CSF motion only
CSF-STREAM	High-resolution CSF mobility near cerebral vessels	Non-invasive spatial characterization of perivascular CSF mobility	Healthy-control study; fluid mobility rather than solute transport; relationship to clearance remains unvalidated	Spatially resolved perivascular CSF dynamics
Fast functional MRI or magnetic resonance encephalography	Temporal coupling of neural/hemodynamic signals with CSF inflow (BOLD–CSF coupling)	Whole-brain physiological timing; sleep-compatible	Indirect; vascular and respiratory sensitivity; coupling is not flux	Neurovascular–CSF coordination
DTI-ALPS	Directional water diffusivity ratio near lateral ventricles	Non-invasive; widely available; retrospective use	Crossing fibers and white-matter geometry; region-of-interest dependence; limited tracer agreement	Perivascular-related diffusion proxy, not clearance
Perivascular spaces, free-water, choroid-plexus, or parasagittal structural MRI	Morphology or water-compartment burden	Anatomical context; scalable	Slow-changing and non-specific; multiple vascular/tissue determinants	Structure, not transport
Wearable impedance + electroencephalography	Time-resolved change in parenchymal electrical resistance	Naturalistic, repeated, high temporal resolution; MRI validation	Global signal; proprietary/investigational; solute transport not measured	Physiological state linked to extracellular volume and exchange

BOLD, blood-oxygen-level-dependent; CSF, cerebrospinal fluid; CSF-STREAM, cerebrospinal fluid-Selective T_2_-prepared REadout with Acceleration and Mobility-encoding; DTI-ALPS, diffusion tensor imaging analysis along the perivascular space; MRI, magnetic resonance imaging.

## 5 Interventional evidence in humans

### 5.1 Sleep and sleep loss

Sleep currently has the most convergent human evidence across multiple levels of the proposed clearance pathway, spanning physiological coupling, sleep-related changes in CSF and tissue-water properties, tracer kinetics, contrast-based exchange, and endogenous brain-derived proteins. Foundational rodent work showed increased interstitial space and faster metabolite clearance during sleep or anesthesia than wakefulness (Xie et al., 2013). In humans, slow-wave sleep is accompanied by large, coupled neural, hemodynamic, and CSF oscillations (Fultz et al., 2019). Recent rodent work identifies norepinephrine-mediated slow vasomotion as a major driver during non-rapid-eye-movement sleep (Hauglund et al., 2025), providing a plausible link between arousal architecture and fluid transport rather than a simple asleep-vs.-awake switch. An earlier human MRI study provides additional, although indirect, support for a sleep-related change in the physiological conditions proposed to facilitate glymphatic transport. Demiral et al. (2019) measured slow and fast components of the apparent diffusion coefficient (ADC) during sleep and wakefulness in 50 healthy adults. Sleep was associated with increased slow ADC in the cerebellum and left temporal pole, decreased fast ADC in several subcortical and paralimbic regions, and an increase in total brain CSF volume. Importantly, the magnitude of the sleep-related increase in cerebellar slow ADC was positively associated with the increase in CSF volume, while greater sleep arousal density was associated with smaller ADC changes. These findings are consistent with the hypothesis that sleep alters extracellular water and CSF-related physiology in a manner that could facilitate glymphatic transport. However, the mixed direction of ADC changes and the absence of direct tracer or endogenous-solute measurements prevent the study from demonstrating enhanced brain clearance. The study therefore supports the mechanistic plausibility of sleep-dependent changes in the neurofluid environment, rather than providing direct evidence of increased solute clearance (Demiral et al., 2019). Most recently, Levitt et al. (2026) provided direct mechanistic and causal evidence that sleep slow waves directly drive human neurofluid dynamics. By implementing phase-targeted closed-loop auditory stimulation synchronized with sleep slow-wave peaks during real-time MRI, the authors demonstrated that amplifying electrical slow waves elicits widespread hemodynamic oscillations and significantly increases CSF flow waves. However, this paper constitutes experimental evidence for modulation of CSF dynamics, without implying increased endogenous solute clearance or clinical benefit (Levitt et al., 2026).

The most direct human experiment used serial intrathecal tracer MRI. Total sleep deprivation reduced overnight tracer clearance from multiple brain regions, and subsequent recovery sleep did not fully normalize the delayed kinetics within the study window (Eide et al., 2021). The study was not a population-based trial and involved participants already undergoing CSF investigations, but its within-protocol tracer tracking directly addresses molecular movement. The randomized crossover biomarker study published in 2026 adds an independent transport chain from brain to plasma and again supports a sleep-active process (Dagum et al., 2026a,b). Together, these experiments justify the conclusion that acute sleep state can modify human brain solute clearance.

What remains unresolved is which component of sleep is necessary and therapeutically tractable. Delta power, microarousals, heart rate, vascular compliance, circadian phase, and prior sleep pressure are correlated and may have different effects. Circadian regulation of glymphatic and lymphatic flow has been demonstrated experimentally (Hablitz et al., 2020), while longitudinal human sampling is rarely standardized for time of day. A useful intervention may need to preserve the oscillatory structure of non-rapid-eye-movement sleep, not simply increase total sleep time.

### 5.2 Sleep disorders and circadian intervention

Obstructive sleep apnea (OSA) combines sleep fragmentation, intermittent hypoxia, intrathoracic pressure swings, and vascular dysfunction, making it a biologically informative but highly confounded natural experiment. In a community cohort of 1,110 adults followed for four years, worsening OSA was associated with declining DTI-ALPS, whereas improvement in OSA tracked increases in the index and better visual memory (Lee et al., 2025). The size and longitudinal design are strengths, but the study did not randomize or standardize OSA treatment and did not measure solute clearance. It supports reversibility of perivascular diffusivity, not proof that continuous positive airway pressure restores glymphatic clearance.

A randomized clinical trial of bright light therapy in subthreshold depression reported changes in DTI-ALPS, free water, and related imaging measures (Chen et al., 2026). This is a valuable demonstration that a circadian intervention can modify a multimodal neurofluid phenotype. The endpoint remains indirect, and symptom improvement cannot establish mediation by brain clearance. Future chronotherapy studies should measure dim-light melatonin onset, sleep architecture, autonomic state, and a transport endpoint in the same protocol.

### 5.3 Anesthesia and sedation

Anesthesia and sedation are important physiological confounders when interpreting glymphatic-related measurements because they alter arousal state, vascular tone, respiration, and CSF dynamics (Hablitz and Nedergaard, 2021; Xie et al., 2013). Preclinical works have shown that anesthesia can modify CSF influx and brain fluid transport (Hablitz et al., 2019; Tong et al., 2026; Xie et al., 2013), while human studies indicate that anesthetic state also changes CSF signal fluctuations and coupling between gray-matter activity and CSF In healthy volunteers, sevoflurane anesthesia induces marked changes in CSF signal dynamics and neurofluid–gray matter coupling, underscoring the direct impact of general anesthetics on human neurofluid physiology (Zimmermann et al., 2025). Recent animal experiments further indicate that different anesthetic regimens, including α_2_-adrenergic agonists, differentially modulate tracer influx (Tong et al., 2026). Human evidence remains limited, and studies of dexmedetomidine, with or without pharmacological support of blood pressure, suggest that sedative-induced changes in CSF dynamics may depend on accompanying cardiovascular and arousal effects (Dagum et al., 2026a,b). These observations are particularly relevant because many experimental studies of glymphatic transport use anesthetized animals, making direct comparison with awake humans difficult. Accordingly, anesthesia or sedation should be treated as an experimental state that can modify the measured phenotype rather than as a neutral background condition. Changes observed under anesthesia should not be interpreted as evidence of enhanced endogenous solute clearance unless transport has been directly demonstrated and alternative effects of altered arousal, respiration, hemodynamics, and vascular tone have been addressed.

### 5.4 Exercise

Regular exercise can alter arterial compliance (Tanaka et al., 2000), autonomic tone (Stein et al., 1999), inflammation (Gleeson et al., 2011), sleep (Kredlow et al., 2015), and metabolic health (Hawley et al., 2014), all of which may influence neurofluid transport (Iliff et al., 2013; Paszkowycz et al., 2025; Zou et al., 2024). The most informative human study separated a single exercise bout from a 12-week cycle-ergometer program. Among 16 completers in the long-term group, putaminal contrast-based influx increased, as did MRI measures interpreted as parasagittal meningeal lymphatic size and flow; efflux and cortical influx did not show corresponding changes (Yoo et al., 2025). A single bout in 21 participants did not produce the same pattern.

This study offers genuine human target-engagement evidence, but the sample was small and young, multiple imaging endpoints were examined, and no inactive randomized control group isolated training from repeated imaging or time effects. The biological signal was region- and phase-specific rather than a global increase in clearance. The appropriate conclusion is therefore that sustained aerobic training may modify contrast-based brain and meningeal fluid exchange. Whether it accelerates removal of endogenous proteins or improves clinical outcomes through that pathway is unknown.

### 5.5 Respiratory manipulation

Inspiration is a major regulator of human CSF movement, acting through craniospinal pressure gradients and venous outflow (Dreha-Kulaczewski et al., 2015; Kollmeier et al., 2022). A 2025 study used real-time velocity-encoding MRI in 20 adults trained in a slow, lower-abdominal breathing practice and 25 untrained controls. Voluntary slow deep breathing increased CSF displacement and net flow, especially at the foramen magnum; trained participants also showed larger movement during regular breathing (Lim et al., 2025). The within-participant breathing manipulation supports causality for CSF motion, while group differences related to long-term training remain susceptible to selection and lifestyle confounding. No parenchymal exchange or solute endpoint was measured.

Intermittent hypercapnia provides a more direct test of vasomotion. In older healthy adults and participants with Parkinson disease, repeated brief carbon-dioxide exposures elicited oscillatory cerebrovascular responses and CSF inflow; the response was attenuated in Parkinson disease (Erhardt et al., 2025). After three 10-min sessions, plasma concentrations of α-synuclein, neurofilament light, glial fibrillary acidic protein, amyloid-β40/42, and phosphorylated tau 217 increased. The combination of induced vasomotion, CSF inflow, and a downstream protein signal is provocative. However, the plasma increase could also reflect altered production, hemoconcentration, blood–brain barrier permeability, or peripheral release; long-term safety and clinical benefit were not tested.

These studies demonstrate that respiratory behavior can modify large-space CSF dynamics in awake humans. They do not justify therapeutic breathing or carbon-dioxide exposure for brain “detoxification.” Intermittent hypercapnia requires controlled gas delivery and monitoring, and sustained hypercapnia may have different vascular effects from oscillatory exposure. A replication trial should include sham gas, arterial or end-tidal carbon dioxide, plasma-volume correction, barrier markers, paired CSF–plasma sampling where feasible, and a prespecified safety framework.

### 5.6 Neuromodulation, light-based, low-intensity ultrasound and surgical interventions

Three small human studies have examined transcranial magnetic stimulation with DTI-ALPS. In insomnia disorder, a prospective study included 28 patients receiving active repetitive transcranial magnetic stimulation (rTMS), nine receiving sham stimulation, and 20 healthy controls. Active treatment improved sleep measures and increased DTI-ALPS relative to sham (Gao et al., 2025). A separate single-arm study treated 22 of 32 patients with chronic insomnia with 10 sessions of low-frequency rTMS; DTI-ALPS and several cognitive measures improved at three months, but the absence of a sham arm prevents attribution to stimulation (Zhang et al., 2025). In 36 older adults with mild cognitive impairment, a sham-controlled crossover protocol found that continuous and intermittent theta-burst stimulation modified DTI-ALPS particularly in APOE ε4 carriers, with exploratory correlations to memory (Sundman et al., 2025).

Together, these studies indicate that a diffusion phenotype can change after brain stimulation. They cannot determine whether stimulation directly changes perivascular transport, acts indirectly through sleep or vascular tone, or changes the white-matter diffusion tensor for unrelated reasons. The strongest next study would combine sham-controlled stimulation with polysomnography or electroencephalography (EEG), vascular measures, and a contrast or solute-transport endpoint. DTI-ALPS should remain secondary until its mediation by actual transport is shown.

Other device-based strategies remain preclinical for clearance. Cervical vagus nerve stimulation increased CSF penetrance in rodents (Cheng et al., 2020). Positive airway pressure increased CSF flow and glymphatic transport in spontaneously breathing anesthetized rodents (Ozturk et al., 2023), but a comparable human target-engagement trial is lacking. Very low-intensity ultrasound (VLIUS) has also emerged as a potential method for modifying glymphatic-related transport. In mice, a 5-min exposure to 1-MHz VLIUS increased CSF tracer influx into paravascular spaces and reduced the amount of exogenous tracer remaining in brain parenchyma, with increased tracer signal in deep cervical lymph nodes. Mechanistic experiments further implicated TRPV4-dependent AQP4 trafficking in the response (Wu et al., 2024). These findings provide unusually direct preclinical evidence linking an externally applied physical stimulus to paravascular influx and tracer clearance, but the study was conducted in animals and used exogenous tracers rather than demonstrating enhanced clearance of endogenous human brain solutes. Multisensory 40-Hz stimulation accelerated glymphatic amyloid removal in mouse models (Murdock et al., 2024), consistent with earlier mouse data for 40-Hz light (Sun et al., 2024). Photobiomodulation has mechanistic plausibility through vascular and mitochondrial effects, yet published glymphatic claims are dominated by animal work and narrative extrapolation (Salehpour et al., 2022). These interventions should be described as preclinical candidates raher than human glymphatic therapies.

Surgical modulation of cervical lymphatic drainage represents another emerging approach. Cervical lymphovenous anastomosis (LVA) is a microsurgical procedure that connects lymphatic vessels with nearby veins and has been proposed as a means of reducing resistance to cervical lymphatic outflow. Its potential relevance to brain clearance arises from the anatomical connection between meningeal lymphatic drainage and the deep cervical lymphatic system. A recent review proposed cervical LVA as a potential strategy for enhancing downstream drainage of CSF and brain-derived solutes in Alzheimer disease, and discussed preliminary clinical experience and candidate outcome measures including imaging, fluid biomarkers, and cognitive testing (Yen et al., 2026). However, the available evidence remains insufficient to establish that LVA increases glymphatic transport or net clearance of endogenous brain solutes. In particular, the currently available literature includes feasibility and outcome reports rather than controlled studies demonstrating a causal chain from altered cervical lymphatic outflow to increased parenchymal solute clearance. LVA should therefore be regarded as an experimental surgical strategy targeting the downstream drainage component of brain-fluid clearance rather than as an established glymphatic therapy.

### 5.7 Posture and other commonly proposed strategies

Lateral sleeping position is frequently presented to the public as a way to improve brain clearance. The foundational experiment showed posture-dependent tracer transport in anesthetized rodents, with lateral positioning outperforming supine or prone conditions (Lee et al., 2015). Human studies show that posture changes venous and craniospinal CSF dynamics, but no controlled human study has demonstrated greater parenchymal solute clearance from a prescribed sleep position. Anatomy, sleep-disordered breathing, anesthesia, and species differences make direct translation inappropriate. Position should therefore be treated as a clear human evidence gap.

Nutritional supplements, hydration, sauna, massage, and commercial “detox” programs have an even weaker evidentiary basis. Diet can influence vascular, metabolic, inflammatory, and sleep pathways, but that does not establish a glymphatic mechanism (Ko and Jeon, 2026; Ota et al., 2025). The same caution applies to pharmacological agents: anesthetics and adrenergic drugs alter arousal and transport in animals, yet clinical dosing must be judged by overall benefit and harm, not by a surrogate clearance signal (Lohela et al., 2022). No nutritional or pharmacological intervention currently has replicated human evidence of enhanced parenchymal solute removal.

## 6 Discussion

### 6.1 Synthesis and interpretation

The first robust conclusion is that human brain-clearance physiology is not fixed, with sleep providing the most convergent evidence across mechanistically distinct human measurements. Sleep alters neural, vascular, and CSF oscillations, and Demiral et al. (2019) further demonstrated state-dependent changes in brain water diffusivity and CSF volume that were spatially and quantitatively related, providing indirect support for the hypothesis that sleep modifies the extracellular-fluid environment relevant to glymphatic transport. More direct evidence subsequently showed that sleep loss changes intrathecal tracer kinetics (Eide et al., 2021), and normal sleep increases overnight transfer of amyloid-β and tau toward plasma in a randomized crossover design (Dagum et al., 2026a,b). This may place sleep above other candidates in both endpoint directness and experimental control. The conclusion is about acute physiology; it does not yet establish that sleep treatment slows neurodegeneration through clearance. Taken together, these findings support sleep as the human intervention domain with the strongest convergence across physiological, tracer-based, and endogenous-solute measurements. However, the Demiral study should not be interpreted as a direct clearance experiment, and the available evidence still does not establish which components of sleep are necessary for enhanced solute removal.

The second conclusion is that several modifiable inputs alter intermediate neurofluid signals. Exercise changes contrast-based exchange and meningeal measures (Yoo et al., 2025). Deep breathing changes CSF movement (Lim et al., 2025). Intermittent hypercapnia changes vasomotion, CSF inflow, and plasma protein signals (Erhardt et al., 2025). Light therapy and TMS change diffusion-based proxies (Chen et al., 2026; Gao et al., 2025; Zhang et al., 2025; Sundman et al., 2025). These are not equivalent levels of evidence, and pooling them under a single label would create false coherence.

The third conclusion is that no intervention has yet completed the translational chain from a prespecified mechanism to a validated transport endpoint and then to a clinical outcome mediated by that transport change. Associations between DTI-ALPS and symptoms or cognition are hypothesis-generating because the metric is biologically non-specific (Georgiopoulos et al., 2024; Schilling et al., 2025; Mossige et al., 2026; Haller et al., 2024; Ringstad, 2024). Likewise, the appearance of proteins in plasma after an intervention is more informative when production, barrier transport, plasma volume, and peripheral sources are measured concurrently.

Table 2 therefore classifies interventions by the actual endpoint reached. Sleep has moderate, convergent human evidence for acute solute transport; exercise has preliminary evidence for contrast-based exchange; respiration has strong evidence for changing CSF motion and early evidence for downstream protein transfer; neuromodulation has preliminary proxy evidence; and posture, vagus nerve stimulation, positive airway pressure, photobiomodulation, and sensory gamma stimulation have no direct human clearance evidence.

**Table 2:** Human and preclinical evidence for modifiable glymphatic-related brain clearance.

Intervention/study	Design and population	Endpoint	Principal finding	Evidence judgment
Normal sleep vs. total sleep deprivation (Eide et al., 2021; Dagum et al., 2026a,b)	Intrathecal tracer cohort; separate randomized crossover, *n* = 39	Tracer clearance; overnight plasma amyloid-β/tau plus modeled physiology	Sleep preserved tracer clearance and increased brain-to-plasma biomarker appearance	Strongest convergent human evidence; acute effects and selected populations limit clinical inference
Sleep vs wakefulness (Demiral et al., 2019)	MRI study, *n* = 50 healthy adults; sleep vs. awake in *n*=30	Slow/fast ADC and CSF volume	Sleep increased CSF volume and produced region-specific ADC changes; cerebellar slow-ADC changes correlated with CSF-volume changes	Mechanistic support for sleep-related neurofluid changes; indirect and not a clearance endpoint
Very low-intensity ultrasound (Wu et al., 2024)	Preclinical mouse study	CSF tracer influx, parenchymal tracer retention, cervical lymph-node tracer signal; TRPV4-AQP4 pathway	VLIUS increased paravascular tracer influx and reduced parenchymal tracer retention, with increased cervical lymph-node tracer signal	Direct preclinical tracer evidence; no human evidence
Cervical lymphovenous anastomosis (Yen et al., 2026)	Review of emerging surgical strategy and preliminary clinical experience in Alzheimer disease	Imaging, fluid biomarkers, cognitive outcomes proposed/reported in early studies	LVA has been proposed to reduce resistance to cervical lymphatic outflow and potentially enhance downstream brain-fluid drainage	Experimental surgical strategy; evidence insufficient to establish increased endogenous-solute clearance
Obstructive sleep apnea trajectory (Lee et al., 2025)	4-year prospective cohort, *n* = 1,110	DTI-ALPS and memory	Improved OSA tracked higher DTI-ALPS and better visual memory; worsening showed the reverse	Large longitudinal association; not randomized treatment and not a clearance endpoint
Aerobic exercise (Yoo et al., 2025)	Single bout (*n* = 21) and 12-week cycling (*n* = 16 completers)	Intravenous contrast T1 mapping; meningeal lymphatic MRI	Long-term, not acute, exercise increased putaminal influx and meningeal measures	Promising target engagement; small, region-specific, no inactive randomized control or solute endpoint
Slow deep breathing (Lim et al., 2025)	Within-person breathing task; trained *n* = 20, untrained *n* = 25	Velocity-encoded CSF motion	Deep breathing increased displacement and net flow, especially at foramen magnum, in both groups	Good evidence for CSF motion; no evidence of parenchymal exchange or clearance
Intermittent hypercapnia (Erhardt et al., 2025)	Healthy older adults and Parkinson disease; three 10-min sessions	Vasomotion, CSF inflow, plasma brain proteins	Induced CSF inflow and increased several plasma protein concentrations, in both groups	Mechanistically coherent but unreplicated; peripheral and barrier explanations remain
Bright light therapy (Chen et al., 2026)	Randomized clinical trial in subthreshold depression	DTI-ALPS, free water, related MRI	Multimodal neurofluid imaging phenotype changed with treatment	Controlled proxy evidence; no direct transport endpoint
Repetitive transcranial magnetic stimulation for insomnia (Gao et al., 2025; Zhang et al., 2025)	Small sham-controlled study; separate single-arm study	DTI-ALPS, sleep, cognition	DTI-ALPS and sleep/cognitive measures improved after treatment	Preliminary; proxy limitations and incomplete sham control
Theta-burst stimulation in mild cognitive impairment (Sundman et al., 2025)	Sham-controlled crossover, *n* = 36	DTI-ALPS and memory	DTI-ALPS increased particularly in apolipoprotein E ε4 carriers; exploratory memory correlations	Hypothesis-generating subgroup effect; no transport endpoint
Posture, vagus nerve stimulation, continuous positive airway pressure, photobiomodulation, and 40-Hz stimulation (Cheng et al., 2020; Ozturk et al., 2023; Murdock et al., 2024; Sun et al., 2024; Salehpour et al., 2022; Lee et al., 2015)	Human clearance intervention absent; mechanistic animal studies	Animal tracer, fluid, or amyloid endpoints	Several positive preclinical signals	Human evidence gap; clinical glymphatic claims are premature

ADC, apparent diffusion coefficient; CSF, cerebrospinal fluid; DTI-ALPS, diffusion tensor imaging analysis along the perivascular space; LVA, lymphovenous anastomosis; MRI, magnetic resonance imaging; TRPV4-AQP4, transient receptor potential vanilloid-4-aquaporin 4.

### 6.2 Clinical implications

Clinicians should continue to diagnose and treat insomnia, OSA, physical inactivity, and vascular risk because their established health consequences and treatment benefits are sufficient. Glymphatic biology may eventually explain part of those benefits, but it is not presently required to justify standard care. Conversely, glymphatic claims should not be used to market unvalidated devices, supplements, sleep positions, breathing programs, or carbon-dioxide exposure.

For research participants and patients, the most accurate language is endpoint-specific. A study may report greater CSF displacement, altered perivascular diffusivity, increased contrast exchange, or higher plasma appearance of a brain-derived protein. The phrase “improved glymphatic clearance” should be reserved for experiments that directly support net solute transport and have excluded major alternative explanations. This linguistic discipline is not semantic caution for its own sake; it determines whether a surrogate can plausibly guide a clinical decision.

### 6.3 Limitations

This is a narrative rather than systematic review. Search and selection were designed to answer a conceptual translational question, not to provide an exhaustive inventory or pooled effect estimate. The literature is evolving quickly, several human studies are small and single-center, and methods differ in anatomy, timing, physiological state, and mathematical assumptions. Our endpoint hierarchy is an interpretive framework, not a formally validated scale.

The term glymphatic-related brain clearance is itself an umbrella. Solute may leave brain through perivascular, transvascular, ventricular, cranial nerve, arachnoid, and meningeal lymphatic routes, and the contribution of each may vary by molecule and state. Intrathecal and intravenous tracers do not behave identically to endogenous proteins. A method that is appropriate for one compartment or disease may not generalize to another. These limitations reinforce the need for endpoint-specific claims rather than negate the evidence of modifiability.

### 6.4 Future perspectives

A next-generation trial should begin with a mechanistically specific intervention and a population in whom the target is plausibly impaired. Examples include cognitive behavioral therapy for insomnia in adults with fragmented non-rapid-eye-movement sleep, supervised aerobic training in sedentary older adults, or optimized OSA therapy in patients with high residual hypoxic or arousal burden. Randomization, allocation concealment, an attention or sham comparator where feasible, and blinded endpoint analysis are essential.

Three endpoint layers should be measured. First, target engagement should quantify the mechanism: polysomnography and arousal architecture for sleep; fitness and arterial compliance for exercise; end-tidal carbon dioxide, diaphragm motion, and autonomic measures for breathing; or electric-field dose and EEG response for neuromodulation. Second, a transport endpoint should capture contrast exchange or a defined solute across at least two compartments. Third, a clinical endpoint should be chosen independently of the clearance hypothesis. Mediation analysis should be prespecified and interpreted only if treatment changes all three layers.

Protocol conditions must be unusually rigorous because neurofluid signals are state-dependent. Time of day, prior sleep, posture, hydration, caffeine, alcohol, medications, blood pressure, heart rate, respiration, carbon dioxide, renal function, and scanner timing can all influence measurements. DTI-ALPS requires harmonized acquisition, reorientation, automated or blinded region placement, crossing-fiber sensitivity analysis, and reporting of the component diffusivities rather than the ratio alone (Taoka et al., 2022; Georgiopoulos et al., 2024; Schilling et al., 2025).

Finally, replication should be built into development. A small mechanistic study can establish target engagement, but a second site or preregistered validation cohort is needed before clinical claims. Shared phantoms, open processing code, common data elements, and negative-control regions would help distinguish a transport signal from scanner, vascular, or white-matter effects. The field will advance faster by invalidating weak surrogates early than by accumulating more disease associations around them.

## 7 Conclusion

Human studies now support a focused answer: glymphatic-related brain-clearance physiology can be modified, but not all reported modifications represent demonstrated clearance. Sleep currently has the strongest convergent human evidence, ranging from sleep-related changes in CSF and tissue-water physiology to intrathecal tracer kinetics and brain-to-plasma transfer of endogenous proteins. Sustained aerobic exercise is promising for contrast-based exchange. Breathing and intermittent hypercapnia can alter CSF and vascular dynamics, with early but unconfirmed evidence of downstream protein transfer. Light therapy and transcranial magnetic stimulation modify imaging proxies whose specificity remains limited. Cervical lymphovenous anastomosis represents an emerging surgical strategy targeting downstream lymphatic drainage, while very low-intensity ultrasound provides promising preclinical tracer evidence; neither currently has sufficient human evidence of enhanced endogenous-solute clearance. Posture, vagus nerve stimulation, positive airway pressure, photobiomodulation, sensory gamma stimulation, nutrition, and pharmacological enhancement lack direct human clearance evidence.

The field's immediate priority is not a larger catalog of candidate therapies. It is a smaller set of rigorously controlled human experiments that connect a mechanism, a validated transport measure, and a meaningful clinical outcome. Until then, standard sleep, respiratory, vascular, and exercise care should be recommended for established indications, while “glymphatic therapy” remains a research concept rather than a clinical category.
